# OP29 Determinants Of The Financial Impact Of Rare Disease Drugs In Italy: Differences Between Expected And Observed Pharmaceutical Expenditure

**DOI:** 10.1017/S0266462324000928

**Published:** 2025-01-07

**Authors:** Francesco Saverio Mennini, Americo Cicchetti, Paolo Sciattella, Filippo Rumi, Matteo Zanuzzi, Angelica Carletto, Annalisa Sammarco, Federica Romano, Pierluigi Russo

## Abstract

**Introduction:**

In Italy, a fixed proportion of health spending is allocated to pharmaceutical expenditure. While the main objective of setting a budget for pharmaceuticals is to control spending, the effectiveness of this ceiling is questionable. This study aims to investigate the determinants of pharmaceutical expenditure for orphan drugs and gather information for effective planning and programming of pharmaceutical spending.

**Methods:**

Data analysis relied on pharmaceutical companies’ pricing and reimbursement (P&R) dossiers submitted to the Italian Medicines Agency (AIFA) for drug-reimbursement approval, along with AIFA’s internal procedural documents. The study encompassed all rare disease drugs reimbursed from January 2013 to January 2019. For each drug, a comparison was made between the expected post-negotiation expenditure and the actual spending observed over the three years following reimbursement approval. Potential determinants of the normalized ratio between observed and expected spending were identified using univariate and multivariate beta regression models. The same methodology was replicated to identify potential determinants of the difference between expected spending before and after negotiation.

**Results:**

Fifty-two rare disease drugs admitted for reimbursement during the study period were analyzed. The median expenditure in the first three commercialization years was 7.6 percent lower than the expected post-negotiation spending. Beta regression analysis indicated a significantly lower reduction for innovative drugs (β 0.736, p-value 0.011 univariate, β 0.585, p-value 0.045 multivariate). Similar effects were observed for P&R procedures (β 0.902, p-value 0.007) and the number of indications presented (β 0.754, p-value 0.021), but only in univariate model. Beta regression analysis for the expected expenditure ratio before/after negotiation revealed a significant effect only for the payment-by-result variable (β 1.485, p-value 0.001).

**Conclusions:**

Observed expenditure for orphan drugs aligns with the expected spending post-negotiation. However, in the subgroup of innovative orphan drugs, the observed pharmaceutical spending was higher than estimated. This could be attributed to prescriber preferences and to a prevalent patient pool awaiting innovative treatment. It appears that the recognition of innovativeness favors orphan drugs that are rewarded with faster market access.

